# Methodology for the Pediatric Dose Optimization for Seizures in Emergency Medical Services (PediDOSE) study

**DOI:** 10.1186/s13063-025-09342-3

**Published:** 2025-12-13

**Authors:** Manish I. Shah, Kathleen M. Adelgais, James M. Chamberlain, Henry E. Wang, Lindsey A. Morgan, James J. Riviello, Rana R. Said, Joseph E. Sullivan, Kimia F. Ghaffari, Kathryn M. Kothari, Mohsen Saidinejad, Robert A. Lowe, Raymond L. Fowler, Catherine R. Counts, Claudia R. Morris, Jonathan R. Studnek, Nancy K. Glober, Caleb E. Ward, Brian M. Clemency, Nicholas Patrick, Rachel D. Munn, Graham M. Brant-Zawadzki, Christian Martin-Gill, Daniel K. Nishijima, Kevin Li, Neomi Sepulveda, John M. VanBuren, The Pediatric Emergency Care Applied Research Network (PECARN) PediDOSE Study Investigators

**Affiliations:** 1 Department of Emergency Medicine, Stanford University School of Medicine, Palo Alto, CA 94304 USA; 2 Section of Pediatric Emergency Medicine, Department of Pediatrics, University of Colorado School of Medicine, Aurora, CO USA; 3 Division of Emergency Medicine, Department of Pediatrics, Children’s National Hospital, The George Washington University School of Medicine and Health Sciences, Washington, D.C. USA; 4 Department of Emergency Medicine, The Ohio State University, Columbus, OH USA; 5 Division of Pediatric Neurology, Department of Neurology, University of Washington, Seattle, WA USA; 6 Division of Neurology, Department of Pediatrics, Baylor College of Medicine, Houston, TX USA; 7 Division of Neurology, Department of Pediatrics, University of Texas Southwestern, Dallas, TX USA; 8 Department of Neurology & Pediatrics, University of California San Francisco, San Francisco, CA USA; 9 Department of Pediatrics, University of Utah, Salt Lake City, UT USA; 10 Division of Emergency Medicine, Department of Pediatrics, Baylor College of Medicine, Houston, TX USA; 11 Department of Emergency Medicine, Harbor — University of California Los Angeles Medical Center, Torrance, CA USA; 12 Columbus Division of Fire, Columbus, OH USA; 13 Division of Emergency Medical Services, Department of Emergency Medicine, University of Texas Southwestern, Dallas, TX USA; 14 Section of Emergency Medical Services, Department of Emergency Medicine, University of Washington, Seattle, WA USA; 15 Division of Emergency Medicine, Department of Pediatrics, Children’s Healthcare of Atlanta, Emory University, Atlanta, GA USA; 16 Wake County Emergency Medical Services, Raleigh, NC USA; 17 Department of Emergency Medicine and Indianapolis Emergency Medical Services, University of Indiana, Indianapolis, IN USA; 18 Department of Emergency Medicine, University at Buffalo, Buffalo, NY USA; 19 Department of Emergency Medicine, Oregon Health and Sciences University, Portland, OR USA; 20 Department of Emergency Medicine, University of Arizona, Tucson, AZ USA; 21 Department of Emergency Medicine, University of Utah, Salt Lake City, UT USA; 22 Department of Emergency Medicine, University of Pittsburgh, Pittsburgh, PA USA; 23 Department of Emergency Medicine, University of California Davis, Sacramento, CA USA; 24 Department of Emergency Medicine, University of California San Francisco, San Francisco, CA USA; 25 Department of Emergency Medicine, McGovern Medical School at the University of Texas Health Sciences Center, Houston, TX USA

## Abstract

**Background:**

Seizures are one of the most common reasons for emergency medical services (EMS) activation for children, and current EMS practice results in underdosing and delayed delivery of anti-seizure medication. A prehospital evidence-based guideline recommends using intranasal or intramuscular midazolam as first-line treatment for pediatric seizures. Despite attempts to implement these guidelines, one-third of children having a paramedic-witnessed seizure have ongoing seizures on emergency department (ED) arrival; this may be due to inadequate or delayed midazolam dosing. Replacing the error-prone, sequential calculations with age-based midazolam dosing may be simpler, faster, and more effective without compromising safety. The objective of this manuscript is to describe the methodology of the Pediatric Dose Optimization for Seizures in EMS (PediDOSE) study, a clinical trial designed to compare the effectiveness and safety of an EMS protocol with four age-based categories for midazolam dosing relative to the current weight-based dosing.

**Methods:**

We are conducting a large EMS-based stepped wedge trial in the Pediatric Emergency Care Applied Research Network (PECARN) by implementing midazolam dosing based on four age categories in seizure protocols in EMS systems in 20 cities. We believe that this implementation will stop more seizures before ED arrival without increasing respiratory failure rates. The primary aim of this study is to compare the effectiveness of age-based EMS midazolam dosing compared to the current weight-based dosing on seizure cessation upon ED arrival. The secondary aim is to determine the frequency of respiratory failure in children after the implementation of EMS midazolam dosing based on these age categories.

**Conclusion:**

If this study demonstrates that an EMS patient care protocol with age-based midazolam dosing is safe and more effective than current practice, the potential impact of this study is a paradigm shift in the treatment of pediatric seizures that can be easily implemented in EMS systems across the country. Beyond seizures, the concept of age-based dosing may also be applicable to other commonly encountered pediatric prehospital conditions for which medication may be indicated.

## Introduction

Emergency medical services (EMS) frequently transport children having seizures, but treatment delays make seizures more difficult to stop and may lead to respiratory failure, neuronal injury, and death [1–5]. Immediate delivery of a therapeutic dose of a benzodiazepine is essential to effectively and safely stop seizures [6]. A pediatric EMS-specific evidence-based guideline (EBG) recommends the initial use of intramuscular (IM) or intranasal (IN) benzodiazepines over other administration routes, considering that obtaining intravenous (IV) or intraosseous (IO) access contributes to delays in medication administration [7]. Evidence also suggests that the rectal (PR) route is inferior to the IM or IN routes [7].

Midazolam is a common benzodiazepine used in EMS based on storage requirements and routes by which it can be administered relative to other benzodiazepines. Though paramedics are more likely to administer the first dose of midazolam via the preferred IN or IM routes after implementing a protocol consistent with the EBG, one-third of patients still arrive at the emergency department (ED) with ongoing seizures, and one-sixth require assisted ventilation [8]. Underdosing and delays in benzodiazepine administration frequently occur and may contribute to treatment failure, while ongoing status epilepticus may contribute to respiratory failure [2, 8]. Scales are not standard equipment on ambulances; rather than obtaining an actual weight, EMS clinicians typically estimate a weight with a length-based tape, calculate a dose in milligrams (mg) using that weight estimate, and then convert that dose to a quantity in milliliters (ml) [9]. Paramedics have suggested eliminating such multiple-step dose calculations and removing protocol ambiguities to improve outcomes [9]. The Rapid Anticonvulsant Medication Prior to Arrival Trial (RAMPART) compared paramedic-administered IV lorazepam and IM midazolam using calculation-free doses in adults and children [10]. RAMPART demonstrated the feasibility of a single benzodiazepine dose in a limited number of children over 2 years old, though a larger pediatric study is needed to demonstrate the effectiveness and safety of simplified dosing in a broader age range of children utilizing both IN and IM routes [10]. Other studies have demonstrated that IN/IM midazolam doses of 0.2–0.5 mg/kg are effective and safe in children in the ED, but no study to date has evaluated age-based dosing categories for both of these routes in children in a prospective prehospital clinical trial [11–13].

The objective of this manuscript is to describe the methodology of the Pediatric Dose Optimization for Seizures in EMS (PediDOSE) study. The aims of PediDOSE are to assess both the effectiveness and safety of using an EMS protocol with four age-based categories for IM/IN midazolam doses in children compared to protocols with weight-based doses for paramedic-witnessed seizures in children. If outcomes are improved, the findings from this study could be easily translated into practice, leading to a paradigm shift in the EMS management of pediatric seizures.

## Methods and analyses

### Study design and setting

The PediDOSE study is a phase 3, multicenter, stepped wedge trial of age-based, paramedic-administered IM/IN midazolam dosing for seizures in pediatric patients in the EMS setting [14–16]. This Exception from Informed Consent (EFIC) study is being implemented by EMS agencies in 20 metropolitan areas across the United States in the Pediatric Emergency Care Applied Research Network (PECARN) [14].

**Fig. 1:**
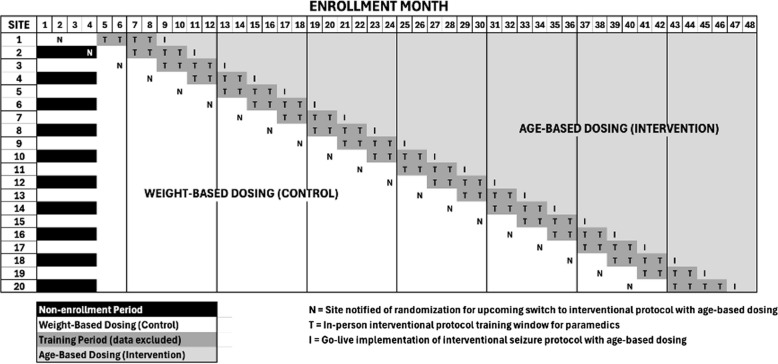
Stepped wedge design and study timeline

Every 2 months during enrollment, the participating EMS agencies in one of the metropolitan areas involved in the study receive a randomization notification and are allowed up to 6 months to both plan and train for the switch from their existing protocol to a protocol with age-based dosing (Fig. 1). Until that randomization notification, allocation is concealed from study investigators, EMS agency collaborators, and the public regarding the timing of when the switch will occur, and each EMS agency is allowed up to 4 months of training prior to implementing the age-based protocol. The age-based interventional protocol replaces the weight-based control protocol, so that only one patient care seizure protocol is in effect at any time. By the end of the 4-year enrollment period, every participating EMS agency will have switched from weight-based to age-based dosing.

We chose not to use individual participant-level randomization to avoid having two potential treatment options in effect on a system-wide level simultaneously. Since paramedics and emergency medical technicians (EMTs) primarily provide prehospital care by following patient care protocols and policies that have been previously approved by an EMS physician, it did not seem feasible to require choosing between two simultaneous patient care protocols. A stepped wedge design allows for staggered implementation of the interventional protocol at all sites.

Anticipating variability in the timing of site completion of the community consultation and public disclosure (CC-PD) process for this EFIC study, the stepped wedge timeline was modified during the CC-PD process to allow a 4-month period of non-enrollment at 10 of the 20 sites. This modification was made to allow sufficient lag time for half of the sites to complete the CC-PD process and obtain Institutional Review Board (IRB) approval to begin enrollment without compromising the overall design. We anticipate that we will enroll up to 6700 participants over this 4-year period.

Hospitals in the metropolitan area of each site are differentiated as being an affiliated ED or nonaffiliated ED. An affiliated ED is an ED where the study team has both full access to the participants’ hospital medical records and investigator capability to monitor for adverse events; nonaffiliated EDs are all other hospitals to which EMS transports eligible patients.

### Participant eligibility

Participants are eligible for the study if they meet all three inclusion criteria: (1) age ≥ 6 months and ≤ 13 years; (2) witnessed by a paramedic to be having a seizure, regardless of seizure type or duration; and (3) transported by an EMS agency participating in this study. Exclusion criteria are as follows: (1) Benzodiazepine allergy, (2) known or presumed pregnancy, or (3) severe growth restriction based on the paramedic’s subjective assessment. Research coordinators verify eligibility through one of four methods: (1) In real time in the ED when the EMS agency transports the child to affiliated EDs; (2) through review of the EMS agency database within 72 h of each transport; (3) through paramedic completion of a study-specific online form, called the Paramedic Self-Report (PSR), after patient handoff in the ED; or (4) through direct voice and/or text contact with research staff available 24 h per day, utilizing a site-specific phone number. Study data were collected and managed using REDCap tools hosted at each site, with the overall study database hosted at the central data coordinating center (DCC) that PECARN utilizes for its studies. REDCap is a web-based, secure software platform that supports data capture for research studies [17, 18].

### Intervention

The timing for the two study arms is shown in Fig. 1. During the control arm, EMS agencies use the weight-based dosing method noted in their EMS protocols. With weight-based dosing, paramedics typically estimate a child’s weight using a commercially available length-based tape; these tapes provide a dose recommendation for medications used in resuscitation, but the doses often do not align with the EMS agency’s protocols that paramedics must follow. As a result, paramedics often use the tape to estimate a weight in kilograms (kg) and then perform sequential calculations to determine the dose in milliliters to administer. The potential routes of administration for midazolam are IM, IN, IO, and/or IV in the EMS seizure protocols during the weight-based dosing period, and the mg/kg dose varies by route.

The EMS protocol with age-based dosing during the interventional arm simplifies the choice of routes from which the paramedic can choose to only IM or IN, standardizes the midazolam concentration to be 5 mg/ml for all EMS agencies, and eliminates all dose calculations. The drug dose is based on the bystander-reported age of the child. If the child’s age is unknown, the paramedic matches the color on the length-based tape to align with one of the four dose options on the study-specific dosing card (Table 1). The midazolam dose is approximately 0.2 mg/kg (range: 0.14–0.26 mg/kg), based on the published EBG for pediatric seizure management [7]. The four dose options ensure the weight-based dose is within 30% of the 0.2 mg/kg EBG-recommended dose based on the 50th percentile weights for age from the Centers for Disease Control growth charts [19]. Upon protocol implementation, a sticker, magnet, card, protocol handbook, online application, and/or other decision support tool will be available on all ambulances. These decision support tools are compatible with what paramedics already access in their EMS agency for dosing guidance and will be located in proximity to where the midazolam is stored, so that the age-based doses for each age category are readily available when drawing up the medication.

**Table 1:** Age-based dosing categories and sequential age de-escalation approach

Ages	Length-based tape colors used to determine dose (if age unknown)	Estimated distribution by age category	Midazolam dose for study intervention
12–13 years^a^	Not applicable (taller than the tape)	8%	10 mg = 2 mL
6–11 years^a^	Green/orange/blue	47%	5 mg = 1 mL
2–5 years^a^	White/yellow	36%	2.5 mg = 0.5 mL
17–23 months^b^	Purple	4%	2.5 mg = 0.5 mL
12–16 months^b^	Red	2%	1.25 mg = 0.25 mL
6–11 months^b^	Pink	3%	1.25 mg = 0.25 mL

^a^Ages and corresponding midazolam doses used when age-based dosing began in year 1 of trial enrollment

^b^Sequential de-escalation of age-based doses contingent upon evaluation of participant safety data

### Age de-escalation

For the age-based doses in the interventional arm, the National Institute of Neurological Disorders and Stroke (NINDS) requested that the investigators use an age de-escalation strategy to balance the need to maximize safety in the youngest pediatric participants while also generating evidence on the effectiveness of age-based dosing in these participants. Therefore, the study was designed to allow age-based dosing for only 2–13-year-old participants initially. When switching to the new patient care protocol, patients less than 2 years old would continue to be dosed according to the weight-based dosing in the EMS agency’s existing patient care protocol with planned, incremental de-escalation of age-based dosing down to 6 months of age contingent upon Data and Safety Monitoring Board (DSMB) approval after evaluating participant safety data on those already enrolled (Table 1). After each anticipated DSMB approval, EMS agencies that subsequently implement the investigational protocol use the revised lower age limit for age-based dosing. All EMS agencies that had previously crossed over to the investigational protocol must begin utilizing the revised lower age limit for midazolam dosing within 12 months of DSMB approval.

### Data collection

The DCC for the study provides data coordination and management services for PECARN and a variety of other national research networks. The DCC developed an electronic data capture system for this study. Data are entered by each clinical site, and data quality is monitored at the DCC. The DCC uses an electronic discrepancy management system to notify sites of inconsistent or erroneous data entry, which is corrected by the clinical site. The discrepancy management system maintains an audit trail of all discrepancy resolutions. Data collected for this study includes EMS records and a PSR for all enrolled subjects. For subjects transported to affiliated EDs, hospital data and rapid response electroencephalogram (RR-EEG) data are also collected. The RR-EEG devices being used for the study at all sites are recorders and headbands manufactured by Ceribell, Inc. (Sunnyvale, CA, USA). Data are collected on all eligible participants during the entire enrollment period, regardless of the status of age de-escalation. Younger participants being treated based on weight-based dosing are part of the control group relative to those being treated under the same investigational EMS treatment protocol that utilizes age-based midazolam dosing for other age groups.

For EMS records, example data variables include dates and times related to the incident (dispatch, scene arrival, scene departure, ED arrival, midazolam administration), vital signs, blood glucose, medications administered by EMS clinicians or bystanders, procedures performed, cardiac rhythms, participant demographics, and past medical history. EMS agencies securely share these data and the EMS clinicians’ narratives with study research staff, so that they can confirm eligibility and enter data into the study’s electronic database for enrolled participants.

A study-specific online PSR was created to capture data that are documented in an inconsistent manner in the EMS record, based on our prior work [8]. Example data variables include confirmation of the inclusion and exclusion criteria, study notification information, details about the paramedic-witnessed seizure(s), medications administered by EMS clinicians or bystanders, response to treatment, method of dose determination, procedures performed, Glasgow Coma Score upon transfer of care in the ED, and paramedic demographics. Paramedics are asked to complete this online self-report as soon as possible after transfer of care to the ED either in-person or online using a REDCap-based form directly accessible in the EMS agency’s electronic health record or by scanning a quick response (QR) code with a phone or tablet.

Hospital data variables collected include the following: Dates and times of key events during the ED visit and/or hospitalization, details about any seizures that occurred in the ED, mental status, vital signs, blood glucose, medications administered, procedures performed, cardiac rhythms, participant demographics, other medical history, weight, disposition, response to treatment, RR-EEG output, and adverse events.

This article is a methodology manuscript, so data sharing is not applicable as no datasets were generated or analyzed to create this manuscript. Since the trial is still enrolling and the timeframe for planned release of the public use dataset has not yet elapsed upon submission of this manuscript, no publicly archived dataset is available for the trial. Data from studies conducted in PECARN are made available in accordance with PECARN policy, and de-identified datasets derived from the completed research protocol will be provided to investigators who agree to follow PECARN’s policies on their use.

### Outcomes

The outcomes of the study are described in Table 2. The study design is novel because it is the first study of status epilepticus that utilizes RR-EEG to assess the primary outcome of seizures on ED arrival with pediatric epileptologists interpreting the waveform output uploaded to the cloud. Prior studies have demonstrated that hospital personnel outside of the ED without prior EEG training can acquire RR-EEG data in a timelier manner than conventional EEG with equivalent quality [20, 21]. While RR-EEG objectively measures the primary outcome, we anticipated that clinical and/or research staff in the ED would face challenges in placing the RR-EEG in some cases or that RR-EEG placement may be delayed. Therefore, the investigators created a pre-specified algorithm for data usage in determining the primary outcome, accounting for the timing of RR-EEG placement, the pediatric epileptologists’ interpretation of the RR-EEG output, whether anti-seizure medication was given prior to RR-EEG placement, and the timing and availability of healthcare professionals’ documented assessments (Table 3).

**Table 2:** Study outcomes

Outcome type	Outcomes
Primary	-Seizing upon emergency department (ED) arrival based on pediatric epileptologists’ interpretation of a rapid response electroencephalogram or physician/nurse/paramedic assessment
Secondary	-Respiratory failure in the prehospital setting or within 30 min of ED arrival, defined as having received bag valve mask ventilation, bi-level positive airway pressure, continuous positive airway pressure, or placement of a supraglottic airway or endotracheal intubation -Time to first midazolam administration after paramedic arrival to the scene
Exploratory	-Time to seizure cessation in the ED, if still having a seizure on ED arrival -Dose/route adherence, defined as receiving the first dose of midazolam within 30% of the recommended dose via a route noted in the protocol that is in effect at the time
Safety	-Life-threatening hypotension within 12 h of ED arrival (based on systolic blood pressure adjusted for age) -Life-threatening cardiac arrhythmia requiring chest compressions, pacing, defibrillation, or medical/electric cardioversion within 12 h of ED arrival -Depressed level of consciousness, defined as Glasgow Coma Score < 8 persisting more than 4 h after ED arrival

**Table 3:** Classification of primary outcome

RR-EEG placed within 15 min of ED arrival	Pediatric epileptologist’s interpretation of RR-EEG	Anti-seizure medication given prior to RR-EEG placement	Clinician judgment	Primary outcome classification
Yes	Seizure	Yes	N/A^b^	Seizure on ED arrival
Yes	Seizure	No	N/A^b^	Seizure on ED arrival
Yes	No seizure	No	N/A^b^	No seizure on ED arrival
Yes	No seizure	Yes	Seizure on ED arrival	Seizure on ED arrival
Yes	No seizure	Yes	No seizure on ED arrival	No seizure on ED arrival
No	N/A^c^	N/A^c^	Seizure on ED arrival	Seizure on ED arrival
No	N/A^c^	N/A^c^	No seizure on ED arrival	No seizure on ED arrival
No	N/A^c^	N/A^c^	Missing	Missing

*ED* Emergency department, *N/A* Not applicable, *RR-EEG* Rapid response electroencephalogram

^a^Based on availability of definitive, documented assessment by the following: (1) first physician or nurse within 15 min of ED arrival, whichever was first, or (2) transporting paramedic if neither physician nor nurse assessment is documented

^b^When RR-EEG is placed within 15 min of ED arrival, the pediatric epileptologist’s interpretation takes priority over clinician judgment if as follows: (1) the epileptologist notes a seizure or (2) when no anti-seizure medication has been given prior to RR-EEG placement and the epileptologist does not note a seizure

^c^When RR-EEG is not placed within 15 min, clinician judgment, if available, takes priority over the pediatric epileptologist’s interpretation

We anticipated that timing would vary from case to case regarding RR-EEG placement relative to administration of anti-seizure medication. This algorithm is designed primarily to rely on the pediatric epileptologist’s interpretation of the RR-EEG to determine the primary outcome when the RR-EEG is placed within 15 min of ED arrival. When the patient receives anti-seizure medication prior to RR-EEG placement, however, the primary outcome classification may not be correct if the pediatric epileptologist does not observe seizure activity. In that case or when RR-EEG placement occurs more than 15 min after ED arrival, clinician judgment is used to determine the primary outcome. It is possible that the primary outcome may be influenced by variable EMS transport times. To account for this, the impact of EMS times for dispatch, scene arrival, midazolam administration, scene departure, and/or ED arrival on the primary outcome will be considered in the final analysis.

At most affiliated sites, a trained individual applies the RR-EEG device to participants, regardless of the randomization status of the EMS agency, unless a parent/guardian objects. The RR-EEG is placed if the participant meets one or more of the following criteria: (1) The participant is actively having a seizure upon ED arrival, based on the parent/guardian’s, physician’s, or nurse’s assessment, or (2) the participant is unresponsive to light touch or voice.

Four pediatric epileptologist co-investigators interpret the RR-EEG output and are blinded to the clinical care that occurs in the ED when making their initial assessment of whether or not the participant is having a seizure on ED arrival; these assessments are not done in real time. For participants who are 6–13 years old, approximately 90% of these RR-EEGs are read by at least one epileptologist; however, at least 10% of these RR-EEGs are read by two epileptologists in order to determine inter-rater reliability. All RR-EEG output on study participants 2–5 years old is read by two epileptologists, since limited validation data for the RR-EEG device exist in this age group. If there is a discrepancy in the reading between these two epileptologists, a third epileptologist makes a tie-breaking determination of the RR-EEG output. When enrollment began, the RR-EEG manufacturer did not yet have Food and Drug Administration (FDA) clearance for a headband to be used in children < 2 years old. Therefore, clinician judgment is the sole method used to classify the primary outcome for participants in this age group.

### Sample size justification

The frequency of seizures on ED arrival was estimated through simulation based on hypothesized frequencies for both weight-based dosing and age-based dosing and based on prior data [8, 22]. The simulations used for calculating the sample size for the stepped wedge design accounted for anticipated site-to-site variability in enrollment. Only randomization sequences that had an expected enrollment size for the weight-based and age-based dosing arms within 5% of each other at the end of the trial were considered. We expect approximately 1210 cases of paramedic-witnessed seizures per year in the participating EMS agencies. Seizure cases that are observed during the training periods noted in Fig. 1 are not counted towards either arm. Accounting for an 8% intra-cluster correlation, up to 10% lack of identification of eligible participants and exclusion of seizures during training periods, we expect to see at least 820 seizure cases per year eligible for analyses. Based on an overall sample size of 4840 participants total utilizing the stepped wedge design as described above, we have approximately 87% power to detect a difference in the rate of seizures on ED arrival between 39% in the weight-based dosing arm and 29% in the age-based dosing arm.

### Ethical considerations

#### Institutional Review Board (IRB) and regulatory approval

The IRB where the DCC for the study is located serves as the single IRB (sIRB). In addition to sIRB activities, each institution has human research protection activities that were completed prior to site activation. Each institution participating in the study, including each of the EMS agencies, was required to file for and comply with the terms of the federal wide assurance (FWA) to adhere to ethical principles of human subjects research and rely upon only IRBs registered with the Office for Human Research Protections. This study is being implemented through an FDA Investigational New Drug application (IND 156119) and is registered on ClinicalTrials.gov (NCT05121324) [14].

#### Exception from informed consent (EFIC)

This study is also being implemented through EFIC (Regulation 21 CFR §50.24) since prehospital seizures are a potentially life-threatening, time-critical condition for which the standard of care is unsatisfactory, obtaining consent in the therapeutic window is not feasible, and there is a prospect of direct benefit for enrolled children [23]. Eligible participants who meet all study eligibility criteria have data collected in accordance with the choice that their parent, guardian, legally authorized representative, or other adult family member has made during the therapeutic time window or at the earliest feasible opportunity for trial notification. Participant assent for enrolled subjects 7-13 years of age is also attempted at the earliest feasible opportunity.

Successful notification is defined as when a parent, guardian, or legally authorized representative informs the research staff about their choice regarding ongoing data collection. In-person study notification is attempted for enrolled participants who are transported to affiliated hospitals. When in-person notification is not feasible or successful, research staff attempt phone notification. When phone notification is not feasible or successful, research staff mail a certified letter to the home address on file in the EMS or hospital record.

One ethical implication of having the intervention be a patient care protocol change is that potential study participants cannot meaningfully opt out of participation in advance since each EMS agency’s weight-based dosing protocol would no longer be in effect. Though the investigators offer parents, legal guardians, and adult family members the option to withdraw from ongoing data collection after they are notified at the earliest feasible opportunity after the participant’s enrollment, it is not possible to opt out of the intervention itself.

Another issue that arose in the design of the trial was how to define the therapeutic window and how to allow for prospective consent during the therapeutic window in the absence of trained research staff in the prehospital setting. For status epilepticus, the investigators defined the therapeutic window as 0–5 min. In accordance with FDA regulations for conducting a study with EFIC, the investigators committed to contacting the parent/guardian and asking for consent within the therapeutic window, if feasible to do so. The sIRB for the study agreed that the paramedics at participating EMS agencies are not classified as being engaged in the research. Therefore, they were not required to provide formal notification about the study during the therapeutic window. They were, however, required to share a 24-h research staff contact phone number with parents and legal guardians during the therapeutic window, as long as it did not compromise patient care. They were also required to note any objections to ongoing participation using the PSR if a parent, legal guardian, or adult family member expressed this desire, such that no further data collection would occur from that point onwards.

#### Safety oversight and reporting

The study has a DSMB composed of individuals independent of the study. Adverse events (AEs) occurring during EMS or hospital care are recorded for eligible participants transferred to an affiliated hospital, up to the time of ED discharge, hospital discharge, or 12 h after ED arrival, whichever is earliest. AEs and serious AEs are categorized by seriousness, expectedness, relatedness, action taken, and outcome.

## Discussion

The methodology for the PediDOSE study is novel in several ways. First, it describes how the effectiveness and safety of age-based categories for EMS medication dosing in children can be studied for a life-threatening condition. Second, the methodology is innovative because it is one of very few EMS studies to utilize EFIC in children. Third, it is one of the first studies to use RR-EEG in the ED to enhance objective assessment of status epilepticus as a study outcome. Finally, the partnership between investigators at EMS agencies and pediatric EDs in 20 metropolitan areas describes a model for generating future evidence specific to pediatric prehospital care.

Pediatric dosing errors are common in EMS for life-threatening conditions such as seizures, cardiac arrhythmias, anaphylaxis, altered mental status, and bronchospasm [24]. In a recent national survey, most paramedics believed that dosing errors could be prevented with a card showing drug doses in milliliters for a specific weight and by eliminating dose calculations [25]. In a separate study, Hoyle et al. also suggested that a specific dose for a range of weights may lead to fewer errors. [24] Implementation of strategies like these in EMS has been primarily limited to retrospective real-world or prospective simulation studies [26–28]. In a before/after observational analysis, Kaji et al. demonstrated fewer epinephrine dosing errors for pediatric out-of-hospital cardiac arrest when paramedics had targeted training on the use of a length-based tape to estimate weight and used precalculated dosing charts [29]. Latimer et al. showed in a prospective cohort study in all ages that EMTs successfully drew up and administered IM epinephrine using an anaphylaxis protocol with two dose options (< 29 kg: 0.15 mg; ≥ 29 kg: 0.3 mg) [30]. Each of these prior studies implemented error mitigation strategies that still relied on weight-based dosing. PediDOSE provides an example of how the effectiveness and safety of a novel age-based dosing strategy to treat a life-threatening condition in children can be studied in EMS with a rigorous prospective design.

PediDOSE is one of very few studies to utilize EFIC in children in the prehospital setting. Though the FDA created the EFIC pathway in 1996, the pathway was not used in a study until a decade later [23, 31]. From 1996 to 2022, 110 trials were either completed, actively recruiting, registered on ClinicalTrials.gov, abandoned before enrollment, or in early planning stages with EFIC in their design [31]. Of the 14 that include children, only 3 noted in the literature target subject enrollment in the prehospital setting: RAMPART, PediDOSE, and the Pediatric Prehospital Airway Resuscitation Trial (Pedi-PART) [10, 31–33]. RAMPART was completed, but it was not pediatric-specific. The PediDOSE and Pedi-PART studies are currently enrolling with anticipated completion in 2026 and 2029, respectively, and both are pediatric-specific [14]. Though EFIC has been rarely utilized for pediatric prehospital research, we showed that among survey respondents in the communities where PediDOSE is enrolling, at least 90% considered PediDOSE an important trial and supported enrollment in their community [32].

The use of RR-EEG to assess the primary outcome of seizure upon ED arrival is unique for a study of status epilepticus. Several randomized trials of anti-seizure medication in children have noted the lack of EEG in the ED as a limitation in definitively assessing whether a seizure had stopped or not [34–37]. Though RR-EEG has been used in other studies in the ED and EMS for status epilepticus, PediDOSE is the only pediatric clinical trial to date to include the use of RR-EEG in the ED to assess the primary outcome [38–41].

Finally, having EMS and pediatric ED-based investigators collaborate together in 20 metropolitan areas across the USA for a clinical trial is unique. Leonard et al. conducted focus groups and interviews of personnel based at EMS agencies affiliated with PECARN [42]. The investigators identified many barriers that decrease and motivators that increase the likelihood of EMS professionals’ participation in research, along with guidance for successful EMS partnerships for pediatric prehospital research [42]. Specifically, thorough planning for research with consideration of the EMS environment, including EMS agency personnel in study planning, clearly defining study procedures with sufficient training about the study, and providing timely feedback to EMS clinicians and the EMS agencies are all tips for ensuring that pediatric prehospital research is successful [42]. Many of these strategies were included in the design and initial implementation of PediDOSE. In particular, the presence of numerous EMS agencies in PECARN who took additional steps to file for FWAs and create data use agreements with academic institutions where research staff were based highlights the importance of having a research network infrastructure to facilitate the conduct of large trials like this.

### Limitations

There are several limitations to the PediDOSE trial design. First is the complexity related to differentiating the presence or absence of a seizure upon ED arrival to classify the primary outcome. Acknowledging that both clinical care and research procedures must occur simultaneously upon ED arrival for patients who had a paramedic-witnessed seizure, the investigators chose to develop a hierarchy incorporating the relative timing in the ED of RR-EEG placement, anti-seizure medication administration, and the first physician and nurse evaluation. We believe this was the most practical solution to avoid a measurement bias while also considering the real-world conditions of implementing a clinical trial in the ED for a life-threatening condition. Another limitation was the logistical challenge posed by requiring sequential DSMB approval for age de-escalation of the age-based midazolam dose. Evidence supports the safety of using IM or IN midazolam doses ranging from 0.2 to 0.5 mg/kg for either status epilepticus in the ED or procedural sedation in the ED or dentist’s offices. Despite this, the investigators agreed to restrict age-based dosing to the 2–13-year-old age group when enrollment began, as the NINDS requested starting with this age group to be consistent with the ages of children enrolled in the RAMPART trial. Finally, the study intervention involved implementation of a bundled patient care protocol that involved several changes: (1) using four age-based categories for midazolam dosing, (2) emphasizing the IM and IN routes as the only first-line options, and (3) prioritizing administration of the first dose of midazolam before checking blood glucose. If there is a difference between the intervention and control groups, it will be difficult to know if it is because one or more of these three changes was impactful in changing the outcome. The pragmatic nature of the intervention and design, however, makes it likely that knowledge can be translated into practice if there is an observed difference between the intervention and control groups.

## Conclusions

We present a study design utilizing EFIC in pediatric patients to study the effectiveness and safety of a patient care protocol change in managing status epilepticus in numerous EMS agencies. If this study demonstrates that age-based midazolam dosing is both safe and more effective than current practice, the potential impact of this study is a paradigm shift that could extend to other medications and conditions in EMS and the ED where complicated calculations are required.

## Data Availability

Data for this study can be found at the Emergency Medical Services for Children Data Center at the University of Utah by contacting Dr. John VanBuren (john.vanburen@hsc.utah.edu). This article is a methodology manuscript, so data sharing is not applicable as no datasets were generated or analyzed to create this manuscript. Since the trial is still enrolling, and the timeframe for planned release of the public use dataset had not yet elapsed upon submission of this manuscript, no publicly archived dataset is available for the trial. Data from studies conducted in Pediatric Emergency Care Applied Research Network (PECARN) are made available in accordance with PECARN policy, and de-identified datasets derived from the completed research protocol will be provided to investigators who agree to follow PECARN’s policies on their use.
